# Associations between maternal urinary kisspeptin in late pregnancy and decreased fetal growth: a pregnancy-birth cohort study

**DOI:** 10.3389/fendo.2024.1257248

**Published:** 2024-01-22

**Authors:** Jiaxian Chen, Lan Yang, Yafei Chen, Wei Yuan, Yao Chen, Hong Liang, Maohua Miao, Gengsheng He, Ziliang Wang

**Affiliations:** ^1^ Shanghai-MOST Key Laboratory of Health and Disease Genomics, NHC Key Lab of Reproduction Regulation, Shanghai Institute for Biomedical and Pharmaceutical Technologies, School of Public Health, Fudan University, Shanghai, China; ^2^ Shanghai-MOST Key Laboratory of Health and Disease Genomics, NHC Key Lab of Reproduction Regulation, Shanghai Institute for Biomedical and Pharmaceutical Technologies, Shanghai, China; ^3^ Department of Nutrition and Food Hygiene, School of Public Health, Key Laboratory of Public Health Safety, Ministry of Education, Fudan University, Shanghai, China

**Keywords:** kisspeptin, neonatal anthropometry, fetal growth, late pregnancy, skinfold thickness

## Abstract

**Background:**

Kisspeptin has been indicated to be a biomarker of fetal growth. Although some evidence suggested that maternal kisspeptin concentrations in early pregnancy were associated with increased fetal growth, studies are still limited and the effect of kisspeptin in late pregnancy remains unknown. This study aimed to investigate the associations between maternal kisspeptin in late pregnancy and fetal growth.

**Methods:**

Based on the Shanghai-Minhang Birth Cohort study, 724 mother-neonate pairs were included in this study. We measured maternal kisspeptin concentrations in the urine samples collected in late pregnancy and neonatal anthropometric indices at birth. The associations between maternal kisspeptin and neonatal anthropometry were investigated using multiple linear regression models.

**Results:**

Higher maternal urinary kisspeptin concentrations were associated with lower neonatal birth weight, head circumference, upper arm circumference, abdominal skinfold thickness, triceps skinfold thickness, and back skinfold thickness. The inverse associations were more pronounced for the highest kisspeptin levels versus the lowest. These patterns were consistent in analyses stratified by neonatal sex, with notably stable associations between maternal kisspeptin concentrations and skinfold thickness.

**Conclusion:**

The present study suggested that maternal kisspeptin concentrations in late pregnancy might be inversely associated with fetal growth. The physiological mechanisms of maternal kisspeptin might differ from those in early pregnancy. Further studies are required to assess associations between maternal kisspeptin and energy homeostasis and explore the physiological roles of kisspeptin in late pregnancy.

## 1 Introduction

Kisspeptin is a family of natural neuropeptides consisting of kisspeptin-54, 14, 13, and 10, encoded by the gene *KiSS-1* (1, 2). It has been proved that kisspeptin is a key regulator of reproductive development and functions, as it stimulates the secretion of Gonadotrophin-Releasing Hormone (GnRH) and activates the Hypothalamic-Pituitary-Gonadal (HPG) axis by binding to its natural ligand G Protein-Coupled Receptor 54 (GPR54), which is encoded by the gene *KiSS-1R* (2).

Recent studies have suggested additional roles of kisspeptin in regulating placentation and later pregnancy. *KiSS-1* and *KiSS-1R* are found highly expressed by trophoblast cells in the placenta during pregnancy (3). With rapid proliferation and differentiation of trophoblast cells, maternal circulating kisspeptin levels continuously increase during pregnancy and peak at delivery (4). Compared with the nonpregnant, circulating kisspeptin levels of pregnant women increase 900-fold in the first trimester and 7000-fold in the third trimester (5). During the initial stages of gestation, kisspeptin plays a key role in inhibiting angiogenesis, restraining trophoblast invasion and migration, and regulating implantation and subsequent placental development (3). Several epidemiological studies have associated decreased maternal kisspeptin levels in early pregnancy with unfavorable pregnancy outcomes, such as spontaneous miscarriage (6), preeclampsia (7), and preterm birth (8). Further, kisspeptin in early pregnancy may also act as a biomarker to predict fetal growth. A few case-control studies have associated maternal kisspeptin levels in the first trimester with increased neonatal birth weight (9–11).

However, growing evidence supports that besides kisspeptin’s important role in regulating early pregnancy, it is also involved in the later fine-tuning of many other key procedures related to fetal growth (11), including maternal energy homeostasis (12) and programming of fetal endocrine functions (13). A study has reported inverse associations between kisspeptin concentrations in late pregnancy and neonatal birth weight, which is inconsistent with the findings for early pregnancy (14). However, another study did not find any associations (15). In general, studies on associations between maternal kisspeptin and fetal growth are still scarce and inconsistent, especially in the lack of evidence of kisspeptin in late pregnancy. Therefore, this study aimed to investigate associations between maternal kisspeptin levels in late pregnancy and fetal growth reflected by a range of neonatal anthropometric indices.

## 2 Materials and methods

### 2.1 Study participants and design

**Figure 1:**
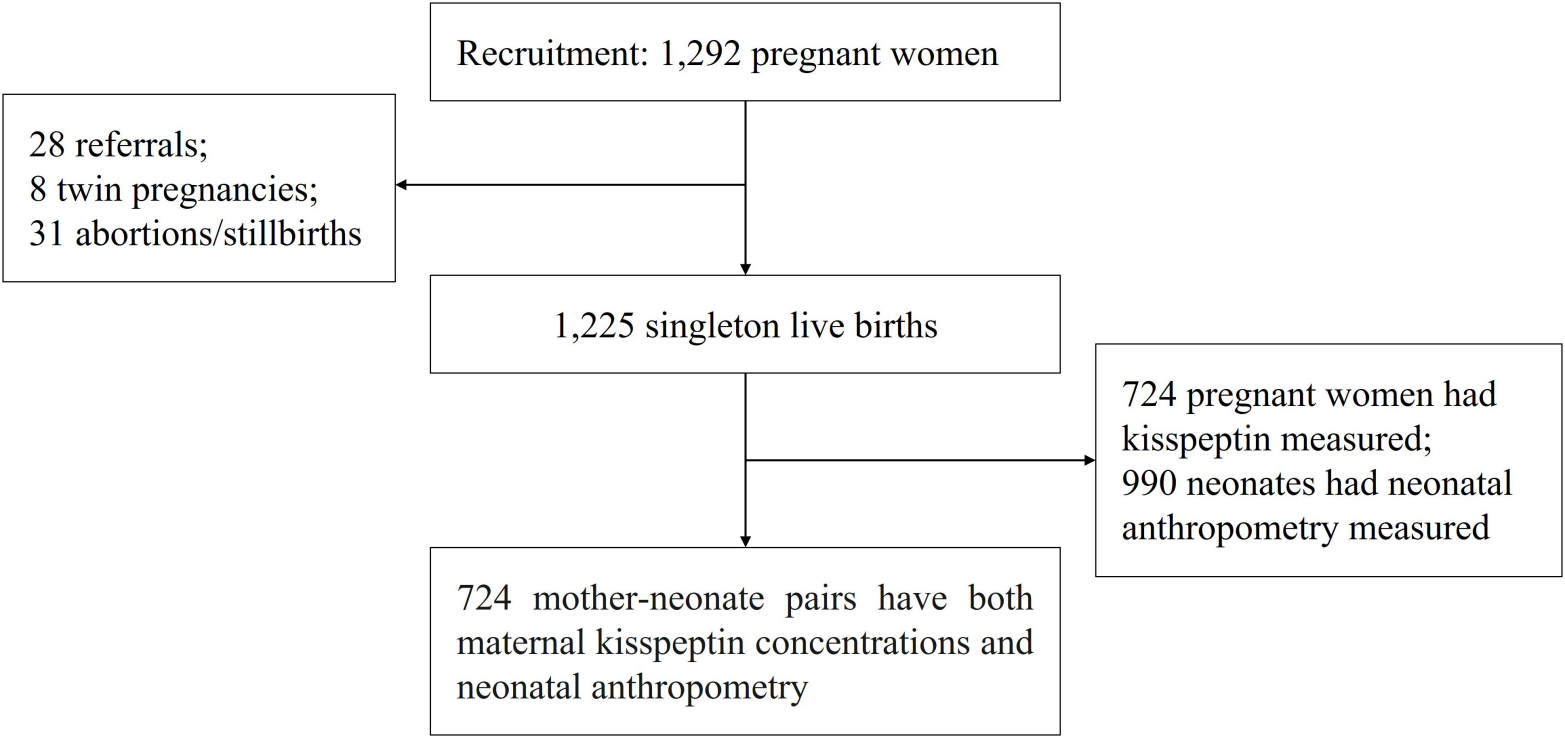
Participant recruitment and flow in the Shanghai-Minhang Birth Cohort Study.

We used data from the Shanghai-Minhang Birth Cohort Study (S-MBCS), which was an ongoing prospective study designed to examine the effects of environmental exposures on both mothers’ and their children’s health. Between April and December 2012, pregnant women were recruited when they visited Minhang Maternal and Child Health Hospital in Shanghai, China for their first prenatal care visit (12-16 weeks of gestation). Detailed inclusion and exclusion criteria have been described elsewhere (16). In total, 1,292 pregnant women were recruited in the study, and 1,233 delivered live infants in the study hospital (28 delivered in another hospital, 31 abortions or stillbirths). We further excluded 8 twin pregnancies, leaving 1,225 live singletons. Single-spot urine samples of pregnant women were collected at 31.6 weeks of gestation on average for kisspeptin measurement. Neonatal anthropometry collection was conducted 1 (with an interquartile range of 1 to 2) day after birth. Due to limited funding, we selected 724 women for urinary kisspeptin measurements, based on criteria such as sufficient urine volume and availability of follow-up visit information (17). Thus, a total of 724 mother-neonate pairs with both maternal kisspeptin concentrations and neonatal anthropometry records were included in this study (**Figure 1**).

All women provided informed consent for themselves and their children at recruitment and each follow-up visit. The study protocol was approved by the ethical committee of the Shanghai Institute for Biomedical and Pharmaceutical Technologies (formerly the Shanghai Institute of Planned Parenthood Research).

### 2.2 Measurement of maternal urinary kisspeptin

Urine samples were collected at 31.6 weeks of gestation on average and frozen at -80°C before being transferred to the laboratory for measurement. Total kisspeptin concentrations (kisspeptin-54, kisspeptin-14, kisspeptin-13, and kisspeptin-10) were measured using the Human Kisspeptin 1(KiSS1) Enzyme-Linked Immunosorbent Assay (ELISA) Kit (Blue Gene, Shanghai, China) according to the manufacturer’s protocol, without any dilution (18). The assay had high sensitivity and excellent specificity according to the introduction of the kit, with inter-assay and intra-assay coefficients of variation<10% and recovery rates ranging from 94% to 103%. More details of kisspeptin measurement have been previously described (19).

### 2.3 Measurement of neonatal anthropometry

We utilized a range of neonatal anthropometric indices to evaluate fetal growth, including birth weight, upper arm circumference, abdominal circumference, head circumference, triceps skinfold thickness, back skinfold thickness, and abdominal skinfold thickness. Each index reflects the growth of distinct body parts. Birth weight, as a comprehensive parameter, is employed to monitor neonatal growth and nutritional status. Head circumference is used as a proxy for brain growth. Upper arm circumference is considered a parameter to reflect the combined muscle and fat. Abdominal circumference is employed to assess the size of the abdominal viscera. Skinfold thickness is utilized to estimate body fat, with upper arm skinfold thickness mainly reflecting peripheral subcutaneous fat and abdominal skinfold thickness mainly reflecting central subcutaneous fat (20, 21).

Neonatal anthropometry collection was conducted 1 (with an interquartile range of 1 to 2) day after birth. The neonate’s birth weight (scaled with fine 1g graduation) was retrieved from the medical birth records (22). Head, arm, and abdominal circumference (scaled with fine 0.1 cm graduation) were measured by tape. Head circumference was measured with the tape placed from above the eyebrows to the maximum protrusion of the occiput. Abdominal circumference was measured with the tape placed around the abdomen just above the umbilicus and perpendicular to the long mid-axis of the trunk. Upper arm circumference was measured with the tape placed through the midpoint between the acromion and tip of the olecranon on the right arm (23). Skinfold thickness (scaled with fine 0.1 mm graduation) was measured using a skinfold caliper. While the neonate was lying in a prone position, physicians stood on the right side of the body and placed the skinfold caliper respectively over the right triceps, midway between the posterior border of the tip of the acromion and the olecranon (triceps skinfold thickness), below the lower angle of the right scapula (back skinfold thickness), and at the intersection point on the midline of the clavicle and parallel to the navel (abdominal skinfold thickness) (24). Each neonate was measured twice by the two proficient physicians who were blinded to the mothers’ kisspeptin levels at the time of measurement, and the averages of their measurements were used for analysis.

### 2.4 Covariates collection

At enrollment, we used a structured questionnaire to collect information about demographic characteristics, reproductive history, health conditions, and lifestyle factors. Maternal pre-pregnancy body mass index (BMI) was calculated as weight (in kilograms) divided by height (in meters) squared. Additionally, maternal urinary creatinine concentration was also measured to control for urine dilution.

### 2.5 Statistical analysis

The included and excluded mother-neonate pairs’ demographic characteristics, pregnancy-related information, and neonatal anthropometric indices were tabulated. We also described the distributions of creatinine-adjusted maternal urinary kisspeptin concentrations among populations with different characteristics. Those data in different groups were compared using student’s t-tests and chi-square tests.

Kisspeptin concentrations were first included as continuous variables in multiple linear regression models to examine the general pattern of associations between maternal kisspeptin concentrations and neonatal anthropometric indices. We further investigated the effects of kisspeptin at different levels by tertiles (the first tertile group as the reference group) in model 2, since generalized additive models (GAM) suggested the existence of nonlinear associations between kisspeptin concentrations and some specific neonatal anthropometric indices. As previous studies had reported sex-specific effects of kisspeptin (12), we further stratified all our analyses by neonatal sex. Covariates were included based on the evidence of a potential confounder from previous literature (22). Additional covariates that changed the estimates by more than 10% were also included. Finally, the following variables were included in multiple linear regression models: maternal age (<25, 25-30 or ≥30 years), maternal education (middle school or below, high school, or college or above), family income per capita (<4000, 4000-8000, or ≥8000 Chinese Yuan (CNY)/month), maternal pre-pregnancy BMI (<18.5, 18.5-24, or ≥24.0 kg/m^2^), paternal drinking before conception (yes or no), maternal weight gain during pregnancy (kg), parity (nulliparous or multiparous), gestational weeks (<37, 37-42, or ≥42 weeks), maternal disease status (yes for mothers with chronic diseases diagnosed before or during pregnancy, such as diabetes mellitus, hypertension, and hypothyroidism, or no) and neonatal sex (boys or girls). Urinary creatinine concentrations were log_10_-transformed and included as a covariate to control for urine dilution (25).

We further conducted several sensitivity analyses to test the robustness of the results. We removed pregnant women with chronic diseases such as diabetes mellitus, hypertension, and hypothyroidism to repeat main analyses among the remaining 499 mother-neonate pairs, considering that these diseases may have effect modifications on the associations between maternal kisspeptin and neonatal anthropometry (9–11). Similarly, we restricted the main analyses to 514 pregnant women whose pre-pregnancy BMI was normal (18.5-24 kg/m^2^) but still included BMI as a continuous variable in linear regression models (10, 26).

All analyses were conducted with SAS version 9.4 (SAS Institute Inc., Cary, NC). A *p*-value less than 0.05 from two-tailed tests was considered statistically significant.

## 3 Results

### 3.1 General characteristics of the population

The characteristics of the participants are presented in **Table 1**. Among the 724 participating mothers, approximately half were between 25-30 years old at parturition (55.11%), reported a monthly family income per capita between 4000-8000 CNY/month (41.76%), and were exposed to passive smoking before conception (40.36%). The majority of participants were well-educated (77.32% graduated from college or above), had a normal weight before pregnancy (72.39% had a BMI between 18.5 and 24 kg/m^2^), did not suffer from chronic diseases before or during pregnancy (68.92%), and reported no alcohol consumption before conception for their partners (68.10%). Compared with the excluded group, pregnant women included were more likely to be nulliparous (86.91% vs. 81.12%), gain more weight during pregnancy (16.56 kg vs. 15.79 kg), and have a longer gestational age (39.61 weeks vs. 39.33 weeks). About 57% of the included neonates were boys, higher than the proportion of excluded male neonates (50.2%). The mean ( ± SD) birth weight, head circumference, upper arm circumference, abdominal circumference, abdominal skinfold thickness, triceps skinfold thickness, and back skinfold thickness of the neonates included were 3442.72 ( ± 432.89) g, 35.16 ( ± 1.18) cm, 11.08 ( ± 1.00) cm, 33.63 ( ± 1.80) cm, 2.63 ( ± 0.76) mm, 4.04 ( ± 1.10) mm, and 3.98 ( ± 1.08) mm, respectively. All anthropometric indices were normally distributed. No significant differences in anthropometric indices were observed between the included and the excluded neonates.

**Table 1:** Characteristics of mother-neonate pairs included and not included.

Characteristics	Included (n=724)	Excluded (n=501)	p^‡^
Maternal age at parturition (years)			0.0837
<25	75 (10.36)	70 (14.62)	
25-30	399 (55.11)	249 (51.98)	
≥30	250 (34.53)	160 (33.40)	
Maternal education			0.3479
Middle school	64 (8.85)	55 (11.00)	
High school	100 (13.83)	75 (15.00)	
College or above	559 (77.32)	370 (74.00)	
Family income per capita (CNY/month)			0.0736
<4000	134 (18.72)	119 (24.14)	
4000-8000	299 (41.76)	190 (38.54)	
≥8000	283 (39.52)	184 (37.32)	
Maternal passive smoking before conception			0.9332
Yes	291 (40.36)	203 (40.60)	
No	430 (59.64)	297 (59.40)	
Paternal drinking before conception			0.7603
Yes	230 (31.90)	163 (32.73)	
No	491 (68.10)	335 (67.27)	
Pre-pregnancy BMI (kg/m^2^)			0.6895
<18.5	138 (19.44)	106 (21.46)	
18.5-24	514 (72.39)	348 (70.44)	
≥24.0	58 (8.17)	40 (8.10)	
Parity			0.0061
Nulliparous	624 (86.91)	404 (81.12)	
Multiparous	94 (13.09)	94 (18.88)	
Gestational age at parturition (weeks)			<0.0001
<37	12 (1.66)	33 (6.60)	
37-42	701 (96.82)	457 (91.40)	
≥42	11 (1.52)	10 (2.00)	
Maternal disease			0.1378
Yes	225 (31.08)	136 (27.15)	
No	499 (68.92)	365 (72.85)	
Total weight gain during pregnancy (kg)	16.56 ± 4.47	15.79 ± 5.11	0.0109
Gestational age at maternal urine collection (weeks)	31.60 ± 1.60	31.77 ± 1.79	0.2941
Neonatal sex			0.0107
Male	417 (57.60)	250 (50.20)	
Female	307 (42.40)	248 (49.80)	
Birth weight (g)	3442.72 ± 432.89	3391.02 ± 546.08	0.1179
Head circumference (cm)	35.16 ± 1.18	35.10 ± 1.18	0.3395
Upper arm circumference (cm)	11.08 ± 1.00	11.17 ± 1.11	0.4789
Abdominal circumference (cm)	33.63 ± 1.80	33.67 ± 1.79	0.7725
Abdominal skinfold thickness (mm)	2.63 ± 0.76	2.62 ± 0.77	0.6157
Triceps skinfold thickness (mm)	4.04 ± 1.10	4.03 ± 1.07	0.9231
Back skinfold thickness (mm)	3.98 ± 1.08	3.88 ± 0.97	0.3075

^‡^p-value for differences between included and not included mother-neonate pairs. BMI, body mass index; CNY, Chinese Yuan.

### 3.2 Distributions of maternal urinary kisspeptin concentrations

The distributions of maternal urinary kisspeptin concentrations are shown in **Supplementary Table 1**. The mean concentration of creatinine-adjusted urinary kisspeptin was 1335.14 ng/g creatinine. Women with higher family incomes had higher kisspeptin concentrations. Additionally, pre-pregnancy BMI was inversely associated with kisspeptin concentrations.

### 3.3 Associations between maternal urinary kisspeptin and neonatal anthropometry

**Figure 2:**
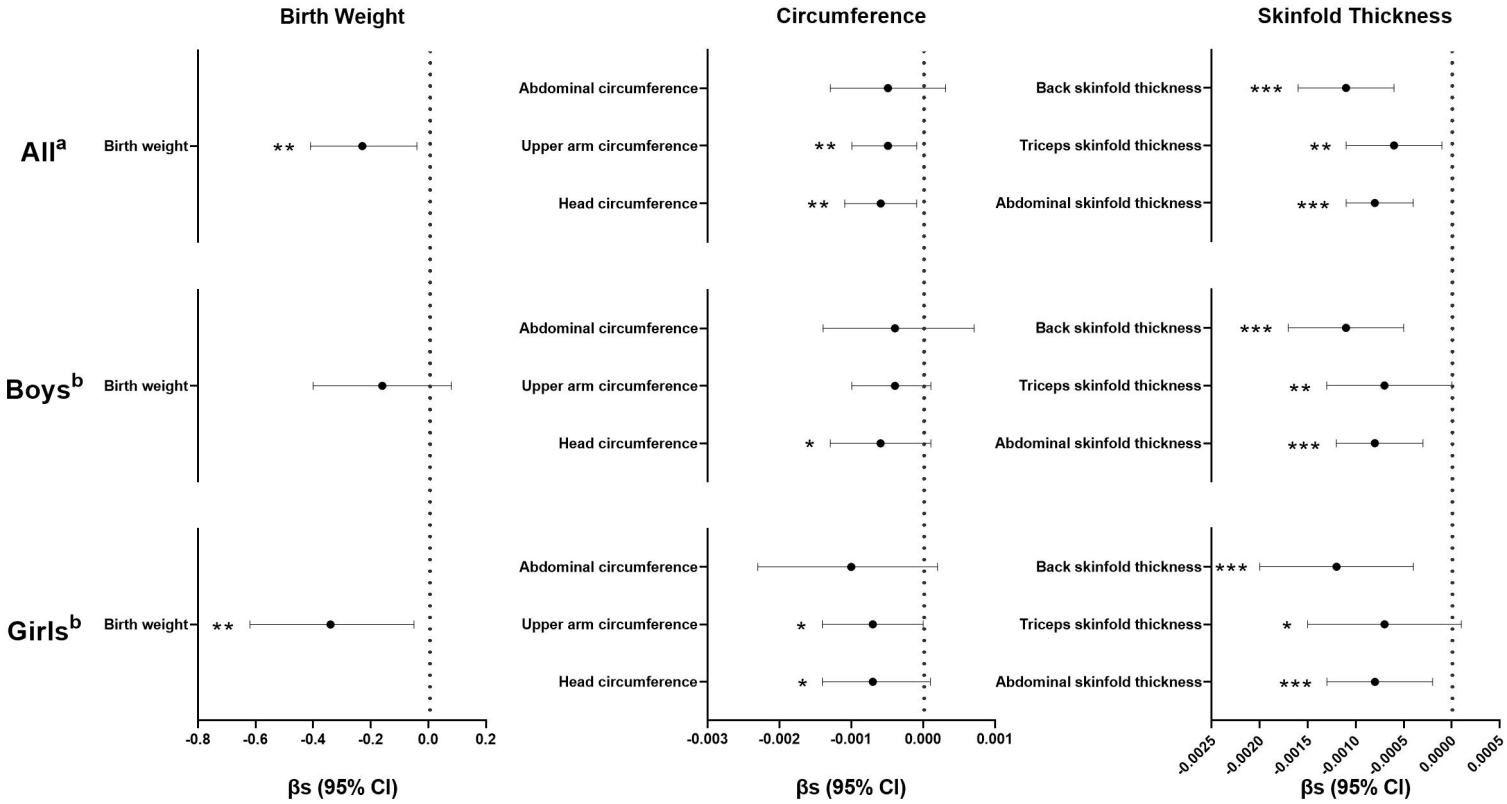
Regression coefficients (βs) and 95% confidence intervals (CIs) for the associations between maternal kisspeptin levels (continuous) in late pregnancy and neonatal anthropometry using multiple linear regression models. ^a^ Adjusted for log_10_-transformed maternal creatinine concentrations, family income, maternal education, paternal drinking before conception, maternal age, maternal pre-pregnancy body mass index, parity, gestational age, total weight gain, maternal disease, and neonatal sex. ^b^ Adjusted for all the variables in Note a except for neonatal sex. Numeric data are available in **Supplementary Table**-**2**. ^*^p< 0.10, ^**^p< 0.05, ^***^p< 0.01, n(All)=724, n(Boys)=417, n(Girls)=307.

In general, inverse associations between maternal urinary kisspeptin concentrations in late pregnancy and neonatal anthropometric indices were observed. In model 1, we used kisspeptin as a continuous variable and found that higher maternal kisspeptin concentrations were consistently associated with lower neonatal anthropometric indices, with statistical significance reaching for all indices except abdominal circumference. When the analyses were stratified by neonatal sex, we found a consistent pattern among both male and female neonates (**Figure 2**; **Supplementary Table 2**).

Further, when kisspeptin was included as a categorical variable in model 2, the inverse associations were mainly found for the highest levels of maternal kisspeptin, compared with the lowest (**Table 2**). Compared with neonates with the first tertile of maternal kisspeptin, those with the highest maternal kisspeptin levels had lower birthweight (β=-88.81, 95% confidence interval (CI): -173.39, -4.24), upper arm circumference (β=-0.21, 95%CI: -0.41, -0.01), abdominal skinfold thickness (β=-0.27, 95%CI: -0.42, -0.11), triceps skinfold thickness (β=-0.25, 95%CI: -0.47, -0.02) and back skinfold thickness (β=-0.40, 95%CI: -0.62, -0.18). Similar patterns were observed in analyses stratified by neonatal sex, with the significance only reached for skinfold thickness. The highest levels of maternal kisspeptin were associated with lower abdominal skinfold thickness (β=-0.23, 95%CI: -0.42, -0.03) and back skinfold thickness (β=-0.34, 95%CI: -0.62, -0.07) among male neonates, and lower abdominal skinfold thickness (β=-0.27, 95%CI: -0.53, -0.01) and back skinfold thickness (β=-0.38, 95%CI: -0.74, -0.01) among female neonates, respectively.

**Table 2:** Regression coefficients (βs) and 95% confidence intervals (CIs) for the associations between maternal kisspeptin levels (categorical) in late pregnancy and neonatal anthropometry using multiple linear regression models^a^.

Neonatal anthropometric indices	Kisspeptin levels	All (n=724)^b^	Boys (n=417)^c^	Girls (n=307)^c^
Birth weight (g)				
	1st tertile	Ref.	Ref.	Ref.
	2nd tertile	4.98 (-70.43, 80.39)	-18.04 (-116.99, 80.92)	14.85 (-102.84, 132.55)
	3rd tertile	-88.81 (-173.39, -4.24)^**^	-72.70 (-182.71, 37.31)	-104.77 (-238.55, 29.02)
Head circumference (cm)				
	1st tertile	Ref.	Ref.	Ref.
	2nd tertile	0.03 (-0.18, 0.23)	0.01 (-0.27, 0.29)	0.00 (-0.31, 0.32)
	3rd tertile	-0.23 (-0.46, 0.00)^*^	-0.24 (-0.55, 0.07)	-0.30 (-0.65, 0.06)
Upper arm circumference (cm)				
	1st tertile	Ref.	Ref.	Ref.
	2nd tertile	0.01 (-0.17, 0.19)	0.01 (-0.22, 0.23)	0.04 (-0.25, 0.34)
	3rd tertile	-0.21 (-0.41, -0.01)^**^	-0.16 (-0.41, 0.09)	-0.24 (-0.57, 0.10)
Abdominal circumference (cm)				
	1st tertile	Ref.	Ref.	Ref.
	2nd tertile	0.12 (-0.21, 0.44)	-0.08 (-0.51, 0.34)	0.31 (-0.20, 0.82)
	3rd tertile	-0.22 (-0.58, 0.15)	-0.29 (-0.76, 0.18)	-0.15 (-0.72, 0.43)
Abdominal skinfold thickness (mm)				
	1st tertile	Ref.	Ref.	Ref.
	2nd tertile	-0.04 (-0.18, 0.10)	-0.05 (-0.23, 0.13)	-0.01 (-0.24, 0.22)
	3rd tertile	-0.27 (-0.42, -0.11)^***^	-0.23 (-0.42, -0.03)^**^	-0.27 (-0.53, -0.01)^**^
Triceps skinfold thickness (mm)				
	1st tertile	Ref.	Ref.	Ref.
	2nd tertile	-0.06 (-0.26, 0.14)	-0.03 (-0.30, 0.23)	-0.01 (-0.33, 0.31)
	3rd tertile	-0.25 (-0.47, -0.02)^**^	-0.14 (-0.44, 0.15)	-0.29 (-0.65, 0.08)
Back skinfold thickness (mm)				
	1st tertile	Ref.	Ref.	Ref.
	2nd tertile	-0.07 (-0.27, 0.12)	-0.02 (-0.27, 0.22)	-0.08 (-0.40, 0.24)
	3rd tertile	-0.40 (-0.62, -0.18)^***^	-0.34 (-0.62, -0.07)^**^	-0.38 (-0.74, -0.01)^**^

a Kisspeptin concentrations were included as a categorical variable by tertiles.

b Adjusted for log_10_-transformed maternal creatinine concentrations, family income, maternal education, paternal drinking before conception, maternal age, maternal pre-pregnancy body mass index, parity, gestational age, total weight gain, maternal disease, and neonatal sex.

c Adjusted for all the variables in Note b except for neonatal sex.

^*^p < 0.10 vs. 1st tertile, ^**^p < 0.05 vs. 1st tertile, ^***^p < 0.01 vs. 1st tertile.

Exclusion of women with chronic diseases or restricting the analyses to women with normal pre-pregnancy BMI did not essentially change the patterns, while several estimates lost statistical significance due to the reduced sample size. Nevertheless, the significant associations between maternal kisspeptin levels in late pregnancy and decreased neonatal fat mass (abdominal skinfold thickness and back skinfold thickness) remained stable (**Supplementary Tables 3, 4**).

## 4 Discussion

To our knowledge, this is the first study to investigate the potential role of maternal kisspeptin levels in late pregnancy in fetal growth reflected by a range of neonatal anthropometric indices, including birth weight, circumference, and skinfold thickness. We observed a consistent pattern of the associations between maternal urinary kisspeptin concentrations in late pregnancy and decreased neonatal anthropometry among both sexes, particularly for the highest kisspeptin levels compared with the lowest. Notably, the significant associations between maternal kisspeptin levels and neonatal fat mass were relatively stable.

Consistent with our results, a case-control study reported the inverse associations between maternal kisspeptin concentrations in late pregnancy and neonatal birth weight among 40 healthy mother-neonate pairs (14). Similarly, inverse associations were also observed in studies on fetal growth and placental expressions of *KISS-1* which are highly correlated with circulating kisspeptin concentrations in late pregnancy since the placenta is the main source of maternal circulating kisspeptin during pregnancy (4, 27). Nevertheless, another case-control study reported no associations between maternal kisspeptin concentrations in any trimester and fetal growth among healthy mother-neonate pairs (15), whose ability to detect statistical significance might be limited by the relatively small sample size (around 25 participants). Notably, the inverse associations of kisspeptin in late pregnancy with neonatal anthropometry in our study were different from those in early pregnancy (9–11, 28).

The different associations between maternal kisspeptin concentrations and neonatal anthropometry could potentially be explained by varying physiological roles and regulatory mechanisms of kisspeptin in different stages of pregnancy. In early pregnancy, placental kisspeptin has been reported to be associated with invasive capacity, with evidence showing that the peak of placental expressions of kisspeptin coincides with implantation and placentation (29). Decreased maternal kisspeptin concentrations in early pregnancy have been linked with impaired fetal growth due to dysfunction of implantation and placentation while administering kisspeptin in early gestation could alleviate the adverse consequences and further improve fetal growth (5, 29–31). Intriguingly, despite the fact that placental expressions of *KISS-1* peak in early pregnancy, circulating kisspeptin concentrations continuously rise until parturition when implantation and placentation have already completed, suggesting that maternal kisspeptin may influence fetal growth through other regulatory mechanisms in later stages of pregnancy (5, 32). Kisspeptin has been suggested to be related to energy homeostasis, with several animal studies showing positive associations between kisspeptin concentrations and energy-regulatory hormones like leptin and oxytocin which can reduce appetite and food intake and increase energy expenditure, especially among pregnant rats (32–35). Recently, kisspeptin has been found to exert direct effects on energy homeostasis (12), supported by the anatomical associations and functional feedback between kisspeptin and key appetite-regulating neurons found in rodent models (36, 37). Both animal and epidemiological studies have reported that lack of kisspeptin leads to increased appetite, body weight, and fat mass (10, 12, 26), which is also in line with the inverse associations between maternal kisspeptin concentrations and pre-pregnancy BMI observed in our study.

Although the physiological mechanisms underlying the interactions between kisspeptin and energy homeostasis are still unclear, the correlations between kisspeptin and energy-regulatory hormones, food intake, body weight, and fat mass may offer some potential explanations for our findings, considering that maternal energy homeostasis is closely linked with fetal growth (34). Moreover, since the placenta has also been supposed to be the main source of neonatal kisspeptin (38), maternal kisspeptin concentrations in late pregnancy may represent the concentrations of neonates. Given the fact that lack of kisspeptin leads to increased body fat mass (39), the stable inverse associations between maternal kisspeptin concentrations and neonatal fat mass observed in our study seemed explicable. In addition, maternal kisspeptin may be also positively associated with fetal leptin levels (13), which provides a further explanation for the inverse associations described above due to leptin’s critical role in reducing body fat (34).

Our study has several strengths. This is the first large-scale prospective study to examine the associations between maternal kisspeptin concentrations in late pregnancy and a range of anthropometric indices, suggesting the roles of kisspeptin in regulating fetal growth in late pregnancy, and thereby offering novel insights. Additionally, we collected a broad range of data on maternal and children’s characteristics, allowing for adjustment for potential covariates. Last but not least, we considered the potential modification effects by neonatal sex, maternal diseases, and maternal pre-pregnancy BMI, and observed similar patterns, indicating the relative robustness of the associations between maternal kisspeptin concentrations and fetal growth.

Despite the strengths, we also have some limitations to acknowledge. Firstly, due to limited funding, our study selected about 60% of mother-neonate pairs for kisspeptin measurement from the cohort. The included and excluded participants had similar characteristics except for the slight differences in gestational age, parity, and maternal weight gain during pregnancy. In addition, no significant differences were found in neonatal characteristics between the included and excluded infants. Thus, our results were less likely to be attributed to selection bias. Secondly, we collected a single-spot urine sample, which may not accurately reflect the kisspeptin concentrations due to the short biological half-life of this biomarker (40). This may lead to non-differential misclassification, resulting in the attenuation of the associations (41). Finally, we did not investigate the interactions between maternal kisspeptin and energy-regulatory hormones in this study. It would be valuable to examine hormones like leptin to explore the mechanisms underlying the associations between maternal kisspeptin and fetal growth in future studies.

In conclusion, this study found that maternal urinary kisspeptin concentrations in late pregnancy were inversely associated with neonatal anthropometry and these associations were mainly found for the highest kisspeptin levels, compared with the lowest. Our results suggest that maternal kisspeptin in late pregnancy may be associated with decreased fetal growth and the related physiological mechanisms may differ from those in early pregnancy. Considering the new findings, further studies are required to corroborate our results. In addition, studies on the potential role of kisspeptin in regulating energy homeostasis are encouraged to explore the physiological mechanisms of the associations we observed.

## Data availability statement

The datasets presented in this article are not readily available because some or all datasets generated may refer to individual privacy. Requests to access the datasets should be directed to ZW, ziliangwang1986@126.com.

## Ethics statement

The studies involving humans were approved by the ethical committee of the Shanghai Institute for Biomedical and Pharmaceutical Technologies (formerly the Shanghai Institute of Planned Parenthood Research). The studies were conducted in accordance with the local legislation and institutional requirements. Written informed consent for participation in this study was provided by the participants’ legal guardians/next of kin.

## Author contributions

JC: Formal Analysis, Writing – original draft, Writing – review & editing. LY: Formal Analysis, Methodology, Writing – review & editing. YFC: Formal Analysis, Methodology, Writing – review & editing. WY: Writing – review & editing, Funding acquisition, Supervision. YC: Formal Analysis, Methodology, Writing – review & editing. HL: Funding acquisition, Methodology, Resources, Writing – review & editing. MM: Funding acquisition, Supervision, Writing – review & editing. GH: Conceptualization, Supervision, Writing – review & editing. ZW: Conceptualization, Funding acquisition, Supervision, Writing – review & editing.
