# Supplementary material for: Associations between maternal urinary kisspeptin in late pregnancy and decreased fetal growth: a pregnancy-birth cohort study
Source: Front Endocrinol (Lausanne). 2024 Jan 22;15:1257248. doi: 10.3389/fendo.2024.1257248 (PMC10839029; doi:10.3389/fendo.2024.1257248)
Supplement: Supplementary file 1 [file Table_1.docx]

Supplementary Material

**Supplementary Table 1. Distribution of creatinine-adjusted urinary maternal kisspeptin concentrations.**

| **Characteristics** | **N** | **Mean ± SD (ng/g creatinine)** | **p-value** |
| --- | --- | --- | --- |
| All subjects | 724 | 1335.14 ± 972.98 |  |
| Maternal age at parturition (years) | | | 0.3276 |
| <25 | 75 | 1262.62 ± 947.04 |  |
| 25-30 | 399 | 1308.93 ± 859.81 |  |
| ≥30 | 250 | 1398.62 ± 1137.13 |  |
| Maternal Education | | | 0.4167 |
| Middle school | 64 | 1193.53 ± 845.62 |  |
| High school | 100 | 1309.05 ± 901.56 |  |
| College or above | 559 | 1356.28 ± 999.44 |  |
| Family income per capita (CNY/month) | | | 0.0039 |
| <4000 | 134 | 1157.29 ± 785.66 |  |
| 4000-8000 | 299 | 1443.85 ± 1047.18 |  |
| ≥8000 | 283 | 1316.08 ± 969.63 |  |
| Maternal passive smoking before conception | | | 0.3451 |
| Yes | 291 | 1324.73 ± 1063.66 |  |
| No | 430 | 1341.46 ± 910.47 |  |
| Paternal drinking before conception | | | 0.2564 |
| Yes | 230 | 1306.36 ± 1040.93 |  |
| No | 491 | 1345.43 ± 941.09 |  |
| Pre-pregnancy BMI (kg/m^2^） | | | 0.0063 |
| <18.5 | 138 | 1431.32 ± 916.62 |  |
| 18.5-24 | 514 | 1343.60 ± 1026.50 |  |
| ≥24 | 58 | 1031.78 ± 593.96 |  |
| Total weight gain during pregnancy (kg) | | | 0.3548 |
| <13 | 138 | 1358.84 ± 915.30 |  |
| 13-19 | 375 | 1304.98 ± 960.66 |  |
| ≥19 | 211 | 1373.10 ± 1032.30 |  |
| Gestational age(weeks) | | | 0.7051 |
| <37 | 12 | 1206.81 ± 928.44 |  |
| 37-42 | 701 | 1338.54 ± 977.27 |  |
| ≥42 | 11 | 1258.50 ± 785.40 |  |
| Parity | | | 0.9542 |
| Nulliparous | 624 | 1320.08 ± 900.73 |  |
| Multiparous | 94 | 1380.54 ± 1082.17 |  |
| Maternal disease | | | 0.5111 |
| Yes | 225 | 1353.61 ± 1147.34 |  |
| No | 499 | 1326.80 ± 884.16 |  |
| Neonatal sex | | | 0.4398 |
| Male | 417 | 1355.76 ± 982.97 |  |
| Female | 307 | 1307.20 ± 960.16 |  |

Notes: *BMI* body mass index; *CNY* Chinese Yuan.

**Supplementary Table 2**. **Regression coefficients (βs) and 95% confidence intervals (CIs) for the associations between maternal kisspeptin levels (continuous) in late pregnancy and neonatal anthropometry using multiple linear regression models^a^.**

| **Neonatal anthropometric indices** | **All (n=724)^b^** | **Boys (n=417)^c^** | **Girls (n=307)^c^** |
| --- | --- | --- | --- |
| Birth weight (g) | -0.23 (-0.41, -0.04)^**^ | -0.16 (-0.40, 0.08) | -0.34 (-0.62, -0.05)^**^ |
| Head circumference (cm) | -0.0006 (-0.0011, -0.0001)^**^ | -0.0006 (-0.0013, 0.0001)^*^ | -0.0007 (-0.0014, 0.0001)^*^ |
| Upper arm circumference (cm) | -0.0005 (-0.0010, -0.0001)^**^ | -0.0004 (-0.0010, 0.0001) | -0.0007 (-0.0014, 0.0000)^*^ |
| Abdominal circumference (cm) | -0.0005 (-0.0013, 0.0003) | -0.0004 (-0.0014, 0.0007) | -0.0010 (-0.0023, 0.0002) |
| Abdominal skinfold thickness (mm) | -0.0008 (-0.0011, -0.0004)^***^ | -0.0008 (-0.0012, -0.0003)^***^ | -0.0008 (-0.0013, -0.0002)^***^ |
| Triceps skinfold thickness (mm) | -0.0006 (-0.0011, -0.0001)^**^ | -0.0007 (-0.0013, 0.0000)^**^ | -0.0007 (-0.0015, 0.0001)^*^ |
| Back skinfold thickness (mm) | -0.0011 (-0.0016, -0.0006)^***^ | -0.0011 (-0.0017, -0.0005)^***^ | -0.0012 (-0.0020, -0.0004)^***^ |

^a^ Kisspeptin concentrations were included as a continuous variable.

^b^ Adjusted for log_10_-transformed maternal creatinine concentrations, family income, maternal education, paternal drinking before conception, maternal age, maternal pre-pregnancy body mass index, parity, gestational age, total weight gain, maternal disease, and neonatal sex.

^c^ Adjusted for all the variables in Note b except for neonatal sex.

^*^p < 0.10, ^**^p < 0.05, ^***^p < 0.01.

**Supplementary Table 3. Regression coefficients (βs) and 95% confidence intervals (CIs) for the associations between maternal kisspeptin levels in late pregnancy and neonatal anthropometry among women without chronic diseases.**

| **Kisspeptin levels** | | **Birth weight** | **Head circumference** | **Upper arm circumference** | **Abdominal circumference** | **Abdominal**  **skinfold thickness** | **Triceps**  **skinfold thickness** | **Back**  **skinfold thickness** |
| --- | --- | --- | --- | --- | --- | --- | --- | --- |
| All (n=499) | | | | | | | | |
| Model 1^a^ | | -0.27  (-0.49, -0.05)^**^ | -0.0006  (-0.0012, 0.0000)^*^ | -0.0006  (-0.0011,  -0.0001)^**^ | -0.0008  (-0.0017, 0.0002) | -0.0008  (-0.0012,  -0.0004)^***^ | -0.0007  (-0.0013,  -0.0001)^**^ | -0.0012  (-0.0017,  -0.0006)^***^ |
| Model 2^a^ | 1st tertile | Ref. | Ref. | Ref. | Ref. | Ref. | Ref. | Ref. |
|  | 2nd tertile | -0.45  (-89.98, 89.08) | -0.01  (-0.26, 0.24) | 0.03  (-0.19, 0.24) | 0.14  (-0.24, 0.52) | 0.001  (-0.17, 0.17) | 0.04  (-0.21, 0.28) | -0.04  (-0.27, 0.19) |
|  | 3rd tertile | -106.00  (-206.20, -5.81)^**^ | -0.29  (-0.57, -0.01)^**^ | -0.19  (-0.43, 0.05) | -0.30  (-0.72, 0.13) | -0.28  (-0.48, -0.09)^***^ | -0.25  (-0.53, 0.02)^*^ | -0.39  (-0.65, -0.13)^***^ |
| Boys (n=294) | | | | | | | | |
| Model 1^b^ | | -0.18  (-0.47, 0.11) | -0.0005  (-0.0014, 0.0003) | -0.0004  (-0.0011, 0.0003) | -0.0005  (-0.0017, 0.0007) | -0.0009  (-0.0014,  -0.0003)^***^ | -0.0007  (-0.0015, 0.0001)^*^ | -0.0011  (-0.0019,  -0.0004)^***^ |
| Model 2^b^ | 1st tertile | Ref. | Ref. | Ref. | Ref. | Ref. | Ref. | Ref. |
|  | 2nd tertile | 9.49  (-106.05, 125.02) | 0.06  (-0.28, 0.40) | 0.09  (-0.18, 0.37) | 0.02  (-0.47, 0.51) | 0.06  (-0.16, 0.28) | 0.18  (-0.13, 0.50) | 0.11  (-0.19, 0.40) |
|  | 3rd tertile | -92.98  (-223.35, 37.39) | -0.30  (-0.68, 0.08) | -0.12  (-0.43, 0.20) | -0.38  (-0.93, 0.17) | -0.23  (-0.47, 0.02)^*^ | -0.08  (-0.44, 0.28) | -0.28  (-0.62, 0.05)^*^ |
| Girls (n=205) | | | | | | | | |
| Model 1^b^ | | -0.46  (-0.80, -0.12)^***^ | -0.0008  (-0.0017, 0.0001)^*^ | -0.0010  (-0.0018,  -0.0002)^**^ | -0.0016  (-0.0030,  -0.0002)^**^ | -0.0008  (-0.0015,  -0.0001)^**^ | -0.0010  (-0.0020,  -0.0001)^**^ | -0.0015  (-0.0024,  -0.0006)^***^ |
| Model 2^b^ | 1st tertile | Ref. | Ref. | Ref. | Ref. | Ref. | Ref. | Ref. |
|  | 2nd tertile | -37.27  (-181.74, 107.20) | -0.14  (-0.52, 0.24) | -0.01  (-0.37, 0.34) | 0.14  (-0.46, 0.74) | 0.00  (-0.29, 0.29) | 0.01  (-0.39, 0.41) | -0.15  (-0.53, 0.23) |
|  | 3rd tertile | -155.84  (-315.59, 3.90)^*^ | -0.44  (-0.86, -0.02)^**^ | -0.33  (-0.72, 0.06) | -0.43  (-1.09, 0.23) | -0.32  (-0.64, 0.01)^*^ | -0.43  (-0.87, 0.01)^*^ | -0.49  (-0.92, -0.07)^**^ |

^a^ Adjusted for log_10_-transformed maternal creatinine concentrations, family income, maternal education, paternal drinking before conception, maternal age, maternal pre-pregnancy body mass index, parity, gestational age, total weight gain, and neonatal sex.

^b^ Adjusted for all the variables in Note a except for neonatal sex.

^*^p < 0.10, ^**^p < 0.05, ^***^p < 0.01.

**Supplementary Table 4. Regression coefficients (βs) and 95% confidence intervals (CIs) for the associations between maternal kisspeptin levels in late pregnancy and neonatal anthropometry among women with normal body mass index.**

| **Kisspeptin levels** | | **Birth weight** | **Head circumference** | **Upper arm circumference** | **Abdominal circumference** | **Abdominal**  **skinfold thickness** | **Triceps**  **skinfold thickness** | **Back**  **skinfold thickness** |
| --- | --- | --- | --- | --- | --- | --- | --- | --- |
| All (n=514) | | | | | | | | |
| Model 1^a^ | | -0.17  (-0.40, 0.05) | -0.0004  (-0.0010, 0.0002) | -0.0004  (-0.0009, 0.0001) | -0.0004  (-0.0013, 0.0006） | -0.0006  (-0.0010,  -0.0002)^***^ | -0.0004  (-0.0010, 0.0002) | -0.0010  (-0.0016,  -0.0004)^***^ |
| Model 2^a^ | 1st tertile | Ref. | Ref. | Ref. | Ref. | Ref. | Ref. | Ref. |
|  | 2nd tertile | 4.20  (-85.47, 93.87) | 0.06  (-0.18, 0.30) | 0.05  (-0.16, 0.26) | -0.09  (-0.47, 0.30) | -0.01  (-0.17, 0.16) | -0.02  (-0.27, 0.22) | -0.02  (-0.26, 0.22) |
|  | 3rd tertile | -40.32  (-140.25, 59.61) | -0.10  (-0.37, 0.17) | -0.08  (-0.31, 0.16) | -0.12  (-0.55, 0.30) | -0.17  (-0.36, 0.01)^*^ | -0.10  (-0.37, 0.18) | -0.27  (-0.54, -0.01)^**^ |
| Boys (n=286) | | | | | | | | |
| Model 1^b^ | | -0.12  (-0.40, 0.15) | -0.0003  (-0.0011, 0.0004) | -0.0002  (-0.0008, 0.0004) | -0.0001  (-0.0013, 0.0011) | -0.0007  (-0.0012,  -0.0002)^***^ | -0.0004  (-0.0012, 0.0003) | -0.0009  (-0.0016,  -0.0002)^**^ |
| Model 2^b^ | 1st tertile | Ref. | Ref. | Ref. | Ref. | Ref. | Ref. | Ref. |
|  | 2nd tertile | -45.21  (-161.48, 71.06) | -0.06  (-0.38, 0.27) | 0.03  (-0.23, 0.30) | -0.45  (-0.95, 0.05)^*^ | -0.08  (-0.29, 0.13) | -0.08  (-0.40, 0.25) | -0.05  (-0.36, 0.25) |
|  | 3rd tertile | -57.68  (-187.67, 72.31) | -0.18  (-0.54, 0.19) | -0.01  (-0.31, 0.28) | -0.23  (-0.79, 0.33) | -0.20  (-0.44, 0.03)^*^ | -0.03  (-0.39, 0.33) | -0.25  (-0.59, 0.09) |
| Girls (n=228) | | | | | | | | |
| Model 1^b^ | | -0.27  (-0.64, 0.11) | -0.0004  (-0.0014, 0.0006) | -0.0007  (-0.0017, 0.0002) | -0.0010  (-0.0026, 0.0006) | -0.0006  (-0.0013, 0.0002) | -0.0004  (-0.0015, 0.0006) | -0.0011  (-0.0022,  -0.0001)^**^ |
| Model 2^b^ | 1st tertile | Ref. | Ref. | Ref. | Ref. | Ref. | Ref. | Ref. |
|  | 2nd tertile | 80.62  (-61.83, 223.06) | 0.20  (-0.16, 0.57) | 0.15  (-0.20, 0.50) | 0.15  (-0.45, 0.75) | 0.08  (-0.19, 0.35) | 0.17  (-0.22, 0.56) | 0.12  (-0.28, 0.51) |
|  | 3rd tertile | -2.22  (-162.63, 158.18) | 0.01  (-0.40, 0.43) | -0.16  (-0.55, 0.23) | -0.05  (-0.72, 0.63) | -0.02  (-0.32, 0.29) | -0.08  (-0.52, 0.36) | -0.12  (-0.56, 0.33) |

^a^ Adjusted for log_10_-transformed maternal creatinine concentrations, family income, maternal education, paternal drinking before conception, maternal age, maternal pre-pregnancy body mass index, parity, gestational age, total weight gain, maternal disease, and neonatal sex.

^b^ Adjusted for all the variables in Note a except for neonatal sex.

^*^p < 0.10, ^**^p < 0.05, ^***^p < 0.01.
